# Organosulfur Compounds in Colorectal Cancer Prevention and Progression

**DOI:** 10.3390/nu16060802

**Published:** 2024-03-11

**Authors:** Patrick L. McAlpine, Javier Fernández, Claudio J. Villar, Felipe Lombó

**Affiliations:** 1 Research Group BIONUC (Biotechnology of Nutraceuticals and Bioactive Compounds), Departamento de Biología Funcional, Área de Microbiología, Universidad de Oviedo, 33006 Oviedo, Spain; mcalpineatsantaclara@gmail.com (P.L.M.); cjvg@uniovi.es (C.J.V.); 2 IUOPA (Instituto Universitario de Oncología del Principado de Asturias), 33006 Oviedo, Spain; 3 ISPA (Instituto de Investigación Sanitaria del Principado de Asturias), 33011 Oviedo, Spain

**Keywords:** allicin, sulforaphane, glucosinolate, indol-3-carbinol, isothiocyanate

## Abstract

This work represents an overview of the current investigations involving organosulfur compounds and colorectal cancer. The molecules discussed in this review have been investigated regarding their impact on colorectal cancer directly, at the in vitro, in vivo, and clinical stages. Organosulfur compounds may have indirect effects on colorectal cancer, such as due to their modulating effects on the intestinal microbiota or their positive effects on intestinal mucosal health. Here, we focus on their direct effects via the repression of multidrug resistance proteins, triggering of apoptosis (via the inhibition of histone deacetylases, increases in reactive oxygen species, p53 activation, β-catenin inhibition, damage in the mitochondrial membrane, etc.), activation of TGF-β, binding to tubulin, inhibition of angiogenesis and metastasis mechanisms, and inhibition of cancer stem cells, among others. In general, the interesting positive effects of these nutraceuticals in in vitro tests must be further analyzed with more in vivo models before conducting clinical trials.

## 1. Introduction

Colorectal cancer (CRC) is one of the most common cancers in Europe after only lung and prostate cancers in incidence in men (incidence of 35–42 cases per 100,000 people) and after breast cancer in women (24–32 cases per 100,000). At the global level, CRC generates 1.85 million new cases and 881,000 deaths annually [1]. The main risk factors associated with this type of cancer are the consumption of alcohol, tobacco, processed meat, saturated fat, and red meat. The increased risk from processed meat consumption is due to its content of nitrosamine-generating preservatives, such as nitrates and nitrites. From red meat consumption, it is due to its high content of heme iron, a cause of oxidative stress in the digestive tract. On the contrary, protective factors include high consumption of whole grains, vegetables and fruits with prebiotic fibers and other nutraceuticals, calcium-rich foods (such as milk), and foods rich in vitamin D [2,3,4]. Some other risk factors include an enrichment in pro-inflammatory gut microbiota taxons, such as *Fusobacterium*, *Porphyromonas*, *Atopobium*, and *Bilophila*, as well as the presence of inflammatory conditions, such as Crohn’s disease [5,6,7]. These pro-inflammatory conditions induce high levels of oxidative stress in the colon mucosa, as well as an impairment in immune response, aberrant cell signaling and upregulation of proliferative pathways, angiogenesis, and migration. A common characteristic under these circumstances is the overexpression of NF-κB, STAT, and HIF1α transcription factors [8,9].

Most CRC cases (about 70%) are due to sporadic mutations in colonocyte genes, associated with chromosomal instability due to *APC* mutations (in addition to mutations in *KRAS*, *TP53*, and other genes), and experience slow progression rates (over 10 years to generate large polyps) [10,11]. The majority of remaining cases of this digestive neoplasia are due to *BRAF* mutation and MAPK activation, giving rise to promoter hypermethylation and gene silencing (such as in *MLH1* or in *p16*). This causes serrated adenomas with crypt branching, flat structure, irregular limits, and a mucus cap that reduces the probability of bleeding. These cases progress rapidly after acquiring microsatellite instability features [12,13]. Surveillance programs (occult fecal blood tests and colonoscopy) reduce the number of advanced tumors, which otherwise may need surgery plus chemotherapy to improve patients’ survival [14].

Some nutraceuticals, such as prebiotic fibers or polyphenols, have demonstrated in vitro and in vivo CRC prevention, by inducing the colonic production of antitumor and anti-inflammatory compounds by the intestinal microbiota, such as short-chain fatty acids (propionate, butyrate) or hydroxycinnamic acids, which block histone deacetylases and promote tumor colonocyte apoptosis [7,15,16,17,18,19,20,21]. These bioactive molecules modulate signaling pathways and the expression of genes involved in apoptosis, cell cycle regulation, and differentiation [15,22]. Another family of plant nutraceuticals are organosulfur compounds, such as diallyl trisulfide (DATS), methylsulfonylmethane, and isothiocyanates. They have been tested as antitumor or chemosensitizers in the co-therapy of CRC, due to their inhibition of matrix metalloproteases, inhibition of carcinogen activation enzymes, and induction of apoptosis [23,24,25]. In the human diet, these organosulfur compounds are present in vegetables such as garlic, onion, broccoli, cabbage, and others from those plant families (*Amaryllidaceae*, *Brassicaceae*) [26,27].

## 2. Organosulfur Compounds Derived from Cruciferous Vegetables

The organosulfur compounds found in cruciferous vegetables (*Brassicaceae* family) can be subdivided into two primary groups, isothiocyanates and indoles. Isothiocyanates can be further divided by those derived from L-methionine and those derived from L-phenylalanine [28,29,30]. The L-methionine-derived isothiocyanates include allyl isothiocyanate (AITC), sulforaphane, sulforaphene, and iberin. The first step in the biosynthesis of these molecules is the variable side-chain elongation of L-methionine [31]. One cycle of elongation gives rise to homomethionine, which then undergoes a series of enzyme-catalyzed reactions to form a desulfoglucosinolate. At this point, the pathway branches into two distinct parts. In one direction, the sulfur atom bound to carbon C4 in desulfoglucosinolate has a carbonyl group added to it and the nitrogen atom has a sulfuric acid added to it in place of the hydroxyl group, forming glucoiberverin. From here, another series of enzyme-catalyzed reactions lead to sinigrin formation, which is the glucosinolate precursor to allyl isothiocyanate (AITC). Upon cell damage, sinigrin comes into contact with the enzyme myrosinase, which hydrolyzes it to form allyl isothiocyanate (AITC) [32]. Glucosinolates are stored in plant cell vacuoles while myrosinase is stored in separate myrosinase cells. It is only upon physical damage to plant tissues that myrosinase is released and comes into contact with glucosinolates to form isothiocyanates [33]. In the second branch of the pathway, the desulfoglucosinolate is converted into glucoiberverin when the sulfur that is bound to carbon C4 has two carbonyl groups added to it and the nitrogen atom has its hydroxyl group replaced with sulfuric acid. Glucoiberverin is then converted into iberin by myrosinase [34].

**Figure 1:**
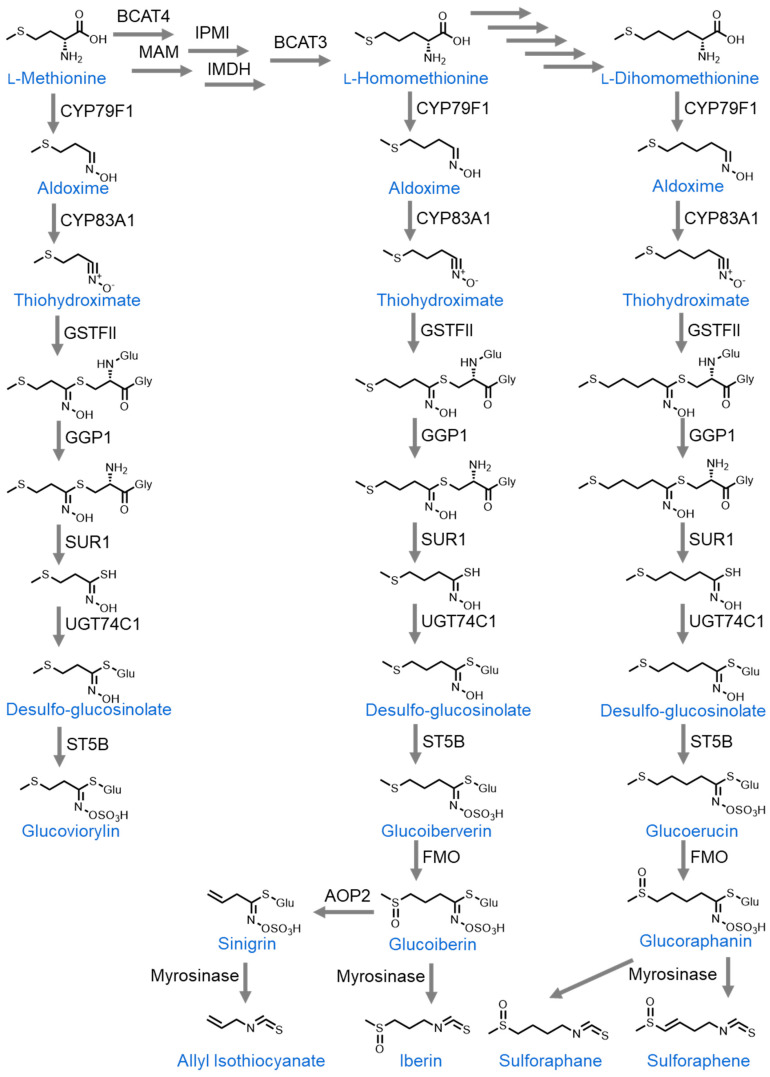
Biosynthesis of glucosinolates from methionine. BCAT4: branched-chain amino transferase 4, MAM: Methylthioalkylmalate synthase, IPMI: Isopropylmalate isomerase, IMDH: Inosine-5′-monophosphate dehydrogenase, BCAT3: branched-chain amino transferase 3, CYP: Cytochrome, GSTFII: Glutathione-S-transferase II, GGP1: Glucosinolate-γ-glutamyl peptidase 1, SUR1: Alkyl-thiohydroxymate C-S lyase, UGT: UDP-glucosyl transferase, ST5B: Aliphatic desulfoglucosinolate sulfotransferase B, FMO: Flavin-containing monooxygenase, AOP2: 2-Oxoglutarate-dependent dioxygenase.

Alternatively, L-methionine can undergo two cycles of side-chain elongation to form dihomomethionine. From there, it is converted to desulfoglucosinolate over the course of multiple reactions. The desulfoglucosinolate then has the hydroxy group on its nitrogen atom replaced with sulfuric acid to form glucoerucin. The sulfur atom on carbon C5 then has a carbonyl group attached to it to form glucoraphanin. Upon cell damage, myrosinase converts glucoraphanin into either sulforaphane or sulforaphene depending on the presence of a double bond between carbons C3 and C4 [33] (Figure 1).

**Figure 2:**
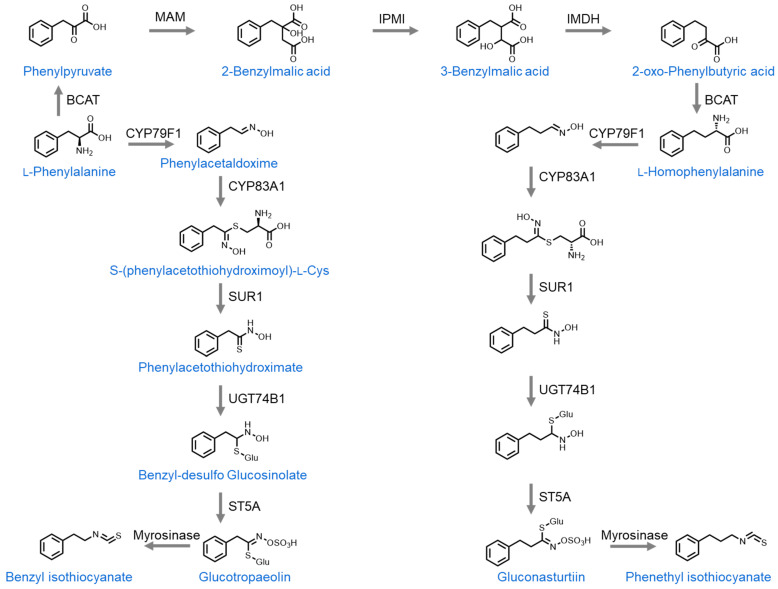
Biosynthesis of glucosinolates from phenylalanine. BCAT: branched-chain amino transferase 4, MAM: Methylthioalkylmalate synthase, IPMI: Isopropylmalate isomerase, IMDH: Inosine-5′-monophosphate dehydrogenase, CYP: Cytochrome, SUR1: Alkyl-thiohydroxymate C-S lyase, UGT: UDP-glucosyl transferase, ST5B: Aliphatic desulfoglucosinolate sulfotransferase B.

The second group of isothiocyanates are those that are derived from L-phenylalanine. Unlike L-methionine, L-phenylalanine does not require side-chain elongation and can directly undergo a series of enzymatic reactions to form benzyl desulfoglucosinolate. This then has the hydroxy group on its nitrogen atom replaced with sulfuric acid to form glucosinolate glucotropaeolin. Upon tissue damage, glucotropaeolin is converted into benzyl isothiocyante (BITC) by myrosinase [35]. Alternatively, L-phenylalanine can undergo one cycle of side-chain elongation to form homophenylalanine. This then undergoes a series of enzymatic reactions to form glucosinolate gluconasturtiin. Upon tissue damage, myrosinase then converts gluconasturtiin into phenethyl isothiocyanate (PEITC) [36] (Figure 2).

**Figure 3:**
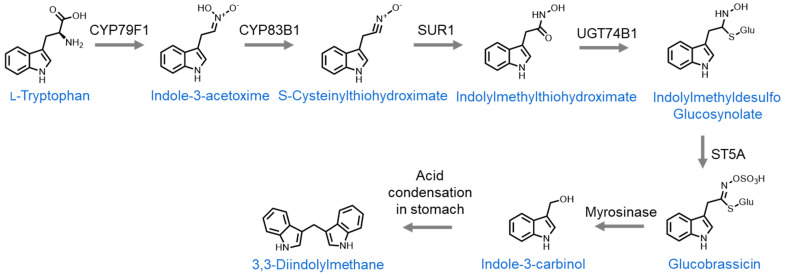
Biosynthesis of indoles from tryptophan. CYP: Cytochrome, SUR1: Alkyl-thiohydroxymate C-S lyase, UGT: UDP-glucosyl transferase, ST5B: Aliphatic desulfoglucosinolate sulfotransferase B.

The third major group of organosulfur compounds found in cruciferous vegetables includes indoles. These are derived from the amino acid L-tryptophan and require no side-chain elongation. Over a series of enzymatic reactions, L-tryptophan is converted into indolylmethyl desulfoglucosinolate. This then has the hydroxy group on the nitrogen atom replaced with sulfuric acid to form glucobrassicin, the glucosinolate precursor to indoles. From here, upon tissue damage, myrosinase converts glucobrassicin to 3-indolylmethylisothiocyanate, which is unstable and hydrolyzes to form indole-3-carbinol [37]. Indole-3-carbinol is itself a potent organosulfur compound, which will be discussed further in this review, but it can also undergo acid condensation in the stomach to form 3,3-diindolylmethane [38] (Figure 3).

### 2.1. Cruciferous Vegetable Consumption

In the last decade, numerous epidemiological studies have indicated the protective effect that cruciferous vegetable consumption has on colorectal cancer development, and this effect has been further confirmed by numerous meta-analyses [39,40,41,42]. The protective effect is still observed when total fiber intake is accounted for, which indicates that this protection comes from molecules found intrinsically in cruciferous vegetables. It is generally regarded that the molecules that induce the antitumoral effect are glucosinolates and their derivatives, indoles, and isothiocyanates. Even so, some epidemiological studies and meta-analyses have not recapitulated these positive results [43,44], indicating that the protective effects of the organosulfur compounds found in cruciferous vegetables may not be readily achieved at dietary concentrations. For this reason, the use of pure compounds and extracts containing glucosinolates at higher, pharmacologically relevant concentrations has been a major area of study.

In addition to the epidemiological studies listed previously, other groups have attempted to confirm the protective effects of cruciferous vegetable consumption in vivo. Studies in rodent models of colorectal cancer have demonstrated the protective effects of including cruciferous vegetables in the diet by detecting a reduced formation of aberrant crypt foci in cruciferous vegetable-eating animals [45,46]. Furthermore, these studies have demonstrated that the protective effects are not recapitulated when other classes of vegetables are included in the diet in place of cruciferous vegetables. With this said, other studies in pigs have demonstrated that broccoli consumption induced detectable levels of nuclear damage in colonic mucosal cells, but that this effect was dependent upon how the broccoli was prepared [47]. Thus, how the vegetables are manipulated may affect their molecular composition, which in turn may modify their impact upon the colorectal epithelium.

In contrast to these animal studies, the consumption of large amounts of cruciferous vegetables has not been found to be protective against colorectal cancer development in human trials. One study included patients who were diagnosed with colonic adenomas and consumed 50 g/day of raw broccoli sprouts every other day for 6 months. They found no statistically significant difference in the numbers of aberrant crypt foci before and after treatment [48]. It should be noted that this investigation took place after CRC diagnosis and thus the protective effects of broccoli consumption prior to CRC development were not investigated.

### 2.2. Cruciferous Vegetable Extracts

Multiple in vitro studies have demonstrated the efficacy of cruciferous vegetable extracts in reducing colorectal cancer cell viability. One study demonstrated that broccoli extract significantly inhibited cell growth in both Caco-2 and HT-29 colorectal cancer cell lines, even more so than pure sulforaphane. The IC 50 of broccoli extract was reached at 1.6 and 3.2 µM of sulforaphane content in each respective cell line, while the IC 50 of pure sulforaphane was reached at 37.5 and 50.9 µM, respectively. This indicates the potential for synergistic effects from multiple antitumoral compounds within the same extract [49]. A second study demonstrated that both broccoli and watercress extracts significantly reduced HT-29 cell viability in the monolayer (IC 50 at 14.8 µM sulforaphane content and 33.9 µM phenethyl isothiocyanate content in broccoli and watercress, respectively) as well as 3D spheroid models (IC 50 at 44.4 µM and 135.6 µM, respectively). This decrease in viability was induced by cell cycle arrest at the G2/M phase. Furthermore, treatment with both extracts decreased the expression of *PROM1* and *LGR5*, two markers of stemness [50]. Thus, cruciferous vegetable extracts may not only reduce cancer cell viability but also reduce the development of cancer stem cells.

In vivo studies in rodents have confirmed the antitumorigenic effects that have been observed in vitro. Kassie et al. showed that the addition of brussels sprouts juices to the drinking water (5% of total volume) of F344 rats in a 2-amino-3-methylimidazo[4,5-*f*]quinoline-induced model of colorectal cancer, starting 5 days before and during cancer induction, significantly reduced the number of colonic aberrant crypt foci 16 weeks after induction. A similar effect was not observed with red cabbage juices at the same concentration [51]. In a similar study by the same group, the addition of brussels sprouts juices to the drinking water of rats in the same model and same concentration, for 25 days, but beginning after tumor initiation, caused a downward trend in the number of colonic aberrant crypt foci, but not a statistically significant decrease at 16 weeks post-induction [52].

A human trial of dietary supplementation with broccoli extract containing 200 µmol of sulforaphane demonstrated a rapid increase in sulforaphane concentration in the participant’s blood plasma (from 0 to 9 µM after 3 h). Supplementation also decreased HDAC3 protein levels and increased p16 protein levels in peripheral blood mononuclear cells (PBMCs) [53]. HDACs (histone deacetylase enzymes) are known to be overexpressed in several cancers and their downregulation is associated with cell cycle arrest and apoptosis [54]. Furthermore, p16 is a well-known tumor suppressor protein and its overexpression is associated with cell cycle arrest. Thus, although broccoli extract has not been demonstrated to directly reduce colorectal cancer development in human trials, it does demonstrate great potential in cancer chemoprevention.

### 2.3. Glucosinolates

#### 2.3.1. Sulforaphane

Of the isothiocyanates derived from cruciferous vegetables, the most studied one in colorectal cancer prevention and treatment is sulforaphane. Numerous in vitro studies in CRC cell lines (Caco-2, HCT116, HT-29, SW480, RKO, DLD-1, LoVo, SW48) as well as patient-derived primary cell cultures have demonstrated its antiproliferative properties via cell cycle arrest, apoptosis, increasing levels of reactive oxygen species, DNA damage, and altered histone acetylation patterns. These effects were achieved at concentrations ranging from 7 to 60 µM depending on the cell type and desired level of toxicity (Table 1) [49,55,56,57,58,59,60,61,62,63,64,65]. Furthermore, treating PBMCs with as little as 5 µM sulforaphane increased their secretion of cytokines and increased the apoptosis of colorectal cancer cells in co-culture [66,67]. Sulforaphane treatment has even been shown to prevent angiogenesis and migration at a concentration of 12.5 µM by inhibiting HIF-1a and VEGF [68]. Finally, sulforaphane has been demonstrated to be cytoprotective of healthy colon cells [47] and to increase the efficacy of other chemotherapeutic agents such as PR-104A (2.5 µM sulforaphane and PR-104A at all concentrations), 5 fluorouracil (7.1 µM of 5 fluorouracil with 5.8 µM sulforaphane derivative), and salinomycin (5 µM salinomycin with 10 µM sulforaphane) (Table 1) [69,70,71]. Sulforaphane at a concentration of 10 µM has also been shown to increase the effectiveness of folinic acid (FOLFOX) against cancer cell lines, but it also increased the expression of multidrug resistance protein 2 (MRP2), meaning that it may reduce the activity of some other chemotherapeutic agents due to their expulsion from the cell [72].

Adding to the in vitro investigations, numerous in vivo studies in murine models have further confirmed sulforaphane’s efficacy in colorectal cancer treatment and prevention. Two recent studies tested sulforaphane’s efficacy against primary CRC cell cultures and Caco-2 cells, and then confirmed their positive in vitro results by testing sulforaphane treatment against xenografts derived from the same primary cells in SCID/nude mice [64,70]. Both studies found that the treatment significantly reduced the size of the xenografts compared with controls, with one study observing a 70% reduction in xenograft size with daily intraperitoneal injections of 0.08 µmoles of sulforaphane (Table 1) [64]. With intraperitoneal administration, these compounds bypass initial structural changes that may occur with oral administration since they are readily absorbed at mesentery capillaries and directed toward the liver via the poral vein. From there, they reenter the digestive tract at the duodenum via bile secretions. Thus, the compounds are protected from modification due to stomach acids and small intestine enzymes, some of which are of microbial origin. For this reason, intraperitoneal injection leads to greater concentrations of the compounds in tissues and plasma prior to possible modifications when compared with oral administration. Furthermore, numerous studies have observed that dietary sulforaphane supplementation at 300 to 400 ppm/day before colorectal cancer initiation significantly reduced the number and size of macroscopic tumors as well as the number of aberrant crypt foci (Table 1) [48,53,73]. These results have been confirmed in APC^min^ mice, azoxymethane-induced CRC mice, and DMH-induced CRC mice and have not displayed any major negative impacts on rodent health.

Shockingly, despite all the promise sulforaphane has shown in CRC treatment and prevention in vitro and in vivo, no clinical trials on the topic have been performed or are in progress as of the writing of this paper, per ClinicalTrials.gov. That said, as of writing, 92 clinical trials have been completed or are in progress testing sulforaphane treatment against a wide variety of other diseases, such as chronic kidney disease, autism, chronic obstructive pulmonary disease, and Parkinson’s disease, indicating its promise for human intake.

#### 2.3.2. Sulforaphene

In contrast to sulforaphane, sulforaphene as a treatment for CRC has been studied far less. One group treated multiple CRC cell lines (HCT116, HT-29, KM12, SNU-1040, and DLD-1) and found that 5 µM sulforaphene was effective in reducing proliferation by arresting the cell cycle at the G2/M phase, upregulating the JNK pathway, inhibiting microtubule polymerization, and increasing intracellular reactive oxygen species. This antiproliferative effect was further confirmed in vivo by observing that daily intraperitoneal injections of sulforaphene at 5 mg/kg of body weight (Table 1) significantly reduced the growth of HCT116 cell-derived xenografts in a nude mouse model [74]. RNA seq analysis of SW480 cell lines treated with sulforaphene or sulforaphane showed remarkable similarity in the gene expression alterations induced by both molecules. In both conditions, genes associated with the p53 signaling pathway, endoplasmic reticulum protein processing, MAPK signaling pathways, and FoxO signaling pathways were overexpressed compared to untreated control cells. Sulforaphene uniquely induced overexpression of genes associated with ubiquitin-mediated proteolysis and estrogen signaling pathways [75]. Considering that the in vitro results were confirmed using multiple cell lines, as well as the similarity in gene expression alterations between sulforaphene and sulforaphane, it seems likely that sulforaphene could have potential uses in CRC treatment.

#### 2.3.3. Allyl Isothiocyanate (AITC)

Allyl isothiocyanate (AITC) has received relatively little attention as a potential treatment for CRC, with only a few in vitro studies published in the last decade and zero in vivo studies. Chiang et al. found that HT-29 cells treated with 20 µM (AITC) exhibited cell cycle arrest at the G2/M phase and increased apoptosis (Table 1). This apoptosis was induced by reactive oxygen species (ROS) related to mitochondrial and endoplasmic reticulum stress. On top of finding increased ROS in the cells, they also found that the growth arrest and DNA damage-inducible protein 153 (DDIT3, also known as CHOP and GADD153) levels were increased, which is a clear marker of endoplasmic reticulum stress. Furthermore, they observed a loss of mitochondrial membrane potential and an increase in cytosolic Ca^2+^, indicating mitochondrial stress [76].

A second study investigating the invasive and migrative capabilities of HT-29 cell lines found that 10 µM AITC treatment inhibited cell migration and invasion via transwell and wound healing assays. Investigation of the protein levels indicated that this inhibition was induced by the downregulation of matrix metalloproteinases 2 and 9, as well as the downregulation of MAP kinases [77]. Thus, AITC shows great potential as a chemotherapeutic agent against CRC, by inhibiting tumor growth and metastasis. Unfortunately, so few studies have been conducted that further investigation is severely needed. The current study only tested the effects of AITC against the HT-29 cell line, so future studies must confirm AITC’s effectiveness against a wider variety of CRC cell lines.

#### 2.3.4. Phenethyl Isothiocyanate (PEITC)

In vitro studies testing phenethyl isothiocyanate (PEITC) against CRC cell lines have demonstrated that PEITC reduces CRC cell viability in the HT-29, DLD-1, and SW480 cell models, while also being effective against CRC stem cells at concentrations from 12 to 88 µM (Table 1) [78,79]. This treatment reduced the number and size of CRC spheroids and appears to be related to Wnt/β-catenin pathway inhibition. Furthermore, Western blot analysis revealed the significant downregulation of proteins associated with stemness, including NANOG, Oct4, and Sox2 [80].

In vivo studies have further confirmed these results. Sprague Dawley rats that had CRC induced by 1,2-dimethylhydrazine (DMH) and received daily intraperitoneal injections of PEITC at 60 mg/kg of body weight developed significantly fewer aberrant crypt foci than untreated controls. Colonic tissue HDAC1, IL-6, and TNF-a levels were also significantly reduced, indicating a reduction in inflammation, and possibly altered histone methylation patterns. In contrast, 20 mg/kg of body weight of 5-fluorouracil (5-FU) administered in the same way was markedly more effective in reducing the numbers of aberrant crypt foci than PEITC and both showed similar levels of hepatotoxicity and nephrotoxicity (Table 1). Interestingly, a combination treatment of low doses of PEITC and the anthraquinone laccaic acid (30 mg/kg and 100 mg/kg, respectively) was even more effective than the 5-FU treatment in reducing the number of aberrant crypt foci while also inducing significantly less hepatotoxicity and nephrotoxicity. Thus, PEITC may have limited use as a sole chemotherapeutic agent due to its inferior performance to 5-FU (the current standard of care), but its use in combination treatments should be further explored [79].

Furthermore, oral PEITC administration at 20 mg/kg of body weight, five times per week for two weeks before HCT116 cell-derived xenograft transplantation in Balb/c nude male mice significantly reduced the xenograft’s growth compared to untreated controls. PEITC treatment beginning after xenograft transplantation also reduced xenograft growth but not as significantly as pretreatment did. Crucially, this was achieved without any negative impact on mouse weight or any induction of hepatotoxicity [80]. Thus, on top of its potential as part of combination treatments, PEITC could have potential use in CRC chemoprevention.

#### 2.3.5. Benzyl Isothiocyanate (BITC)

Benzyl isothiocyanate (BITC) is a compound that has received some attention as a potential chemotherapeutic agent in vitro but has been sparsely investigated in vivo. One study demonstrated that treating a CRC cell line (HCT116) with 20 µM BITC reduced cell viability, induced apoptosis, and induced cell cycle arrest at the sub-G1 phase (Table 1). Interestingly, these effects coincided with the activation of the PI3K/Akt/FoxO pathway. This pathway drives cell growth and proliferation and is associated with oncogenesis, differentiation, and drug resistance. Further experiments combining BITC treatment with the use of PI3K inhibitors demonstrated significantly greater cytotoxicity than BITC alone. For this reason, the authors hypothesize that the observed activation of the PI3K/Akt/FoxO pathway serves as a resistance mechanism for the cell against BITC treatment [81]. Thus, BITC may be of limited use as a chemotherapeutic agent on its own, but it also has the potential to reduce the efficacy of other drugs in combination treatments due to its activation of the PI3K/Akt/FoxO pathway.

Abe et al. found that 10 µM BITC inhibits proliferation by activating p65. When activated, p65 upregulates nuclear factor-κB (NF-κB) transcription and inhibits β-catenin activity. This causes the downregulation of cyclin D1 expression and arrests the cell cycle. Interestingly, BITC’s antiproliferative effects are only observed in HT-29 cells with mutated p53 in this study [82]. BITC’s antiproliferative effects were significantly reduced in HCT116 cells with wild-type p53, contrasting the results of the previously described study.

In sharp contrast to the positive results of the in vitro studies, the in vivo results are much less promising. In a large study of CRC chemoprevention in azoxymethane-induced F344 rats, Wargovich et al. found that BITC dietary supplementation as low as 0.5 g/kg of diet increased the formation of aberrant crypt foci when compared to animals fed a standard diet (Table 1) [83]. Seeing as the positive effects of BITC treatment vary based on the cell line assayed and are p53 mutation-dependent, BITC could have a possible function as a chemotherapeutic agent against CRCs with specific genotypes. The in vivo study demonstrates that BITC is likely not effective as a chemopreventive agent where its function would be to prevent tumorigenesis in healthy cells, but this does not take away from its potential in treating specific CRC genotypes.

#### 2.3.6. Iberin

Iberin is an organosulfur compound that has been studied very little in the context of CRC treatment and prevention. One study found that treating Caco-2 CRC cells with 8 µM iberin resulted in a significant decrease in cell proliferation. DNA methylation analysis found that iberin treatment did not affect p16, ESR1, and LINE-1 methylation patterns but did significantly decrease the percent methylation of methyl guanine methyl transferases (MGMTs). This coincided with an increase in DNMT1, 3, and 3B mRNA levels. The alterations in the methylation patterns were further confirmed in a second CRC cell line (HCT116), although changes in proliferation were not investigated in this line. It is known that DNMT mRNA levels vary depending on the cell cycle stage, which indicates a possible connection between MGMT expression levels and iberin’s antiproliferative effect, but this cannot be confirmed since cell cycle analysis was not conducted in this study [84]. In a separate study, Slaby et al. analyzed the miRNA profiles of non-transformed colonic epithelial cell lines treated with 10 µM iberin (Table 1). They found that total miRNA levels were higher in treated vs. untreated cells, which indicates a potential antitumoral effect since global downregulation of miRNA levels has been observed in CRC tumors. Furthermore, treatment induced an increase in miR-23b levels. miR-23b is a known tumor suppressor that regulates the epithelial–mesenchymal transition and TGF-β signaling [85]. In both studies, iberin and sulforaphane were compared, with very few differences between their effects. As such, iberin may have some potential as a chemotherapeutic compound against CRC, but a great deal of research remains to be conducted, especially in in vivo models, which are entirely lacking. Furthermore, the impact of iberin on CRC cell viability should be investigated in a wider variety of cell lines. It could be argued that iberin’s effects on healthy cells’ miRNA levels make it a candidate for chemoprevention.

### 2.4. Indoles

#### 2.4.1. Indole-3-Carbinol

Indole-3-carbinol (I3C) is an organosulfur compound found in cruciferous vegetables and its antitumorigenic properties against CRC have been well characterized both in vivo and in vitro. Treating a wide variety of CRC cell lines (DLD1, HCT116, HT-29, LS513, RKO, LoVo, and SW480) has confirmed its ability to reduce cancer cell viability and proliferation by inducing apoptosis, although with large IC 50s, at 500 µM at 48 h and 1 mM at 24 h [86,87]. Of more promise is the finding that the I3C derivative 3-(2-bromoethyl)-indole had an IC 50 of only 50 µM at 24 h (Table 1) [88]. Mechanistically, one study found that I3C treatment induced the upregulation of p53, which induced apoptosis and inhibited cell migration [65]. Furthermore, a separate study found that I3C is an aryl hydrocarbon receptor (AHR) agonist. Activation of AHR appears to be critical to I3C’s apoptosis-inducing activity since AHR downregulation protected the cells and prevented apoptosis [87].

In contrast to the positive in vitro results, in vivo studies in rodents have yielded mixed results. In one study, rats that were fed hemin (a potential carcinogen in red meat) and had CRC induced by DMH experienced less tumor incidence and growth when I3C was supplemented into their diets at a concentration of 1 g/kg, leading to an average consumption of 23.43 mg/rat/day [89]. At the same time, supplementing the diets with I3C, probiotics, and prebiotics increased the size and incidence of the tumors relative to I3C supplementation alone, indicating potential metabolization by the gut microbiota. In direct contrast to these results, a subsequent study by the same group found that rats with CRC induced by DMH experienced increased tumor incidence and size when I3C was supplemented into their diets at a concentration of 1 g/kg (Table 1). The addition of prebiotics and probiotics to the diet along with I3C canceled out the effects of I3C and gave similar results to the un-supplemented controls [90]. Furthermore, a third study by a different group found that C57BL/6J mice that were infected with *Citrobacter rodentium* and treated with dietary I3C at 1 mmol/kg (0.15 g/kg) of diet experienced reduced colonic *C. rodentium* colonization, reduced crypt hyperplasia, and reduced inflammatory biomarkers IL-17A, IL-6, and IL-1β. They also found that treating Caco-2 cells in culture with 25 µM I3C reduced *C. rodentium* growth and prevented it from binding to the cells [91].

What these three studies have in common is that they attempted to modulate the intestinal microbiomes of the rodent models that they used. It is well known that intestinal dysbiosis is associated with the development of colitis-associated CRC. For this reason, investigation into the effects of any supplement on CRC development should be conducted in the context of the intestinal microbiome. Studies have shown a large amount of variation in the intestinal microbiota between rodents from different vendors and even rodents housed in different cages for prolonged periods of time [128]. This could be one of the reasons for the conflicting in vivo results. Due to the positive in vitro results in numerous cell lines, as well as some positive results in vivo, I3C should be considered a promising chemotherapeutic agent in CRC treatment or prevention. With this said, a more in-depth characterization of the microbiome is necessary to understand its confounding effects.

#### 2.4.2. 3,3′-Diindolylmethane

3,3′-Diindolylmethane (DIM) is an organosulfur compound generated from the dimerization of I3C under the gastric acidic pH that has received quite a bit of attention as a potential treatment for CRC. In vitro studies have been extremely promising and have confirmed DIM’s anticancer properties in a wide variety of cell lines. One study found that treatment of multiple CRC cell lines (HCT116, HT-29, HCT15, and DLD1) with 40 µM DIM reduced their viability and induced cell cycle arrest at the G1 phase (Table 1). This was found to be induced by COX1/2 and ERK1/2 protein inhibition [92]. Furthermore, other studies have confirmed the findings that a wide variety of CRC cell lines (HCT116, SW480, HT-29, LoVo, Caco-2, and Colo-320) showed increased apoptosis, cell cycle arrest, and reduced viability after DIM treatment at 25–56 µM concentrations. (Table 1). These results are attributed to endoplasmic reticulum stress and cyclin D1 downregulation [93], upregulation of the N-Myc pathway [94], ATF-3 upregulation [95], and inhibition of calcium channels [96].

In contrast to the positive in vitro results, in vivo studies have yielded mixed findings. Two studies have demonstrated DIM’s efficacy in reducing the size of cell- and patient-derived xenografts in Balb/C mice. One study found that DIM treatment by intraperitoneal injection of 40 mg/kg of body weight increased the sensitivity of DLD-1 and HCT116 cell-derived xenografts to 5-Fluorouracil treatment, and this was found to be associated with the inhibition of pyrimidine metabolism [97]. A second study found that daily oral DIM treatment at 40 mg/kg body weight significantly reduced the growth of patient-derived xenografts and did so without the induction of any detectable side effects. In contrast to these positive results, this same study also found that daily oral DIM treatment at the same concentration did not reduce the metastatic ability of HT-29 cells injected into the tail vein of Balb/C mice and did not reduce the formation of CRC tumors in APC^min^ mice (Table 1) [92]. Thus, DIM may have some usefulness as a chemotherapeutic agent, but the lack of chemopreventive activity in APC^min^ mice raises serious questions about its usability in more physiologically relevant models. Also, it shows little use in reducing metastasis. Of note is the fact that I3C must undergo condensation to DIM (and also other polymerization products) under the gastric acidic pH in order to obtain bioactivity. This does not occur in the case of intraperitoneal administration, nor in the case of in vitro cell line treatments. Considering that DIM is effective at much smaller concentrations than I3C, it is possible that a large part of I3C’s activity may be due to these polymerization reactions.

## 3. Organosulfur Compounds Derived from *Allium* Species

**Figure 4:**
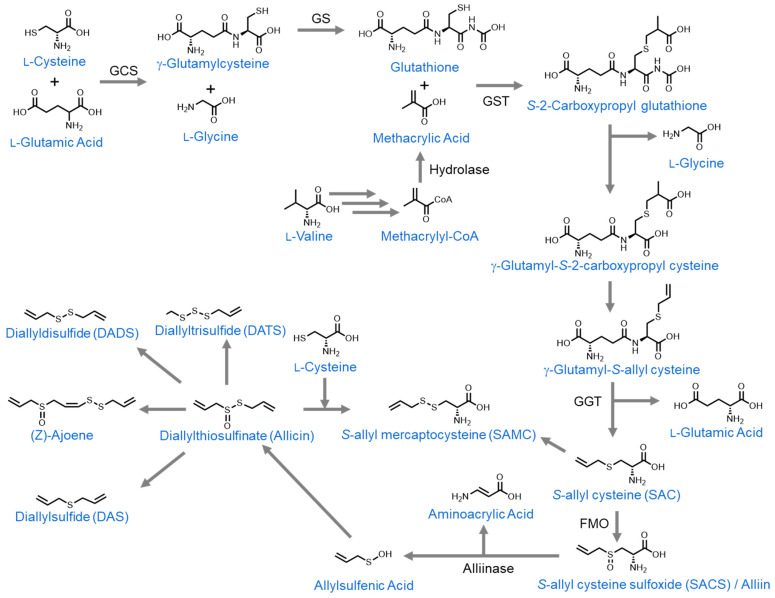
Biosynthesis of organosulfur compounds from allium vegetables. GCS: γ-Glutamylcysteine synthetase, GS: Glutathione synthetase, GST: Glutathione S-transferase, GGT: γ-Glutamyl transferase, FMO: Flavin-containing monooxygenase.

*Allium* is a genus of enormous economic importance, belonging to the *Amaryllidaceae* family. The majority of bioactive organosulfur compounds found in *Allium* vegetables are derived from glutathione, which is formed by a series of reactions between the amino acids L-cysteine, L-glutamic acid, and L-glycine. Glutathione reacts with methacrylic acid, an L-valine derivative, to form S-(2-carboxypropyl)glutathione. This then undergoes a series of reactions to form S-allylcysteine (SAC) [129]. SAC then has a carbonyl group added to its sulfur atom to form S-allylcysteine sulfoxide (SACS), also known as alliin [130]. Upon cellular damage (for example, during garlic crushing before cooking), alliinase is released from the vacuole and comes into contact with alliin. When this occurs, it converts alliin to allylsulfenic acid, which dimerizes to form diallyl thiosulfinate, also known as allicin [131]. Allicin is relatively unstable, so it rapidly rearranges to form numerous derivates. In this review, we will discuss the derivates (Z)-ajoene [132], diallyl sulfide (DAS), diallyl disulfide (DADS), diallyl trisulfide (DATS), and diallyl tetrasulfide [133,134]. Furthermore, during the preparation of aged garlic extract, S-allyl cysteine and diallyl disulfide react to form S-allylmercaptocysteine (SAMC) [135] (Figure 4).

### 3.1. Allium Vegetable Consumption

Epidemiological studies on *Allium* vegetable consumption and colorectal cancer have been promising, although inconclusive. For example, a study in China with 1666 patients found that consumption of *Allium* vegetables (>16 kg/year) was associated with a lower risk of colon cancer, with the exception of distal colon cancer where no association was found [136]. Moreover, a meta-analysis with a sample of 12,558 patients suggested similar effects from the consumption of the *Allium* genus against CRC [137]. In contrast, a meta-analysis of 5458 CRC patients found no association between *Allium* consumption and CRC risk. It even showed that *Allium* consumption was associated with a 23% increase in CRC incidence in women. The authors attribute this contradictory increase to the consumption of a high concentration of flavonoids present in *Allium* vegetables, which may function as an inhibitor of hormonal metabolism and affect estrogen metabolism [138].

Studies of *Allium* consumption and CRC in humans have many limitations, primarily due to experimental variability. The size of the garlic portions taken in the different studies, the presence of multiple food items, the cooking method, the bioactivity of the different *Allium* vegetables, the length of the studies, the heterogeneity of participants, cultural differences between the different regions studied, and the study design are some prominent examples [139]. Thus, further epidemiological and clinical studies that can minimize these variables are required to draw consistent conclusions.

### 3.2. Allium Vegetable Extracts

*Allium sativum* is a vegetable widely used in gastronomy and is commonly known as garlic. The chemical composition of garlic is very broad, highlighting mixtures of soluble organosulfur compounds with different health properties such as antioxidant, antimicrobial, hypoglycemic, antiobesity, or anticancer [140].

Studies conducted by Bagul et al. showed that a crude garlic extract at concentrations of 0.5 µg/mL produced a growth inhibition of greater than 50% in colon cancer cells (Caco-2). They even observed that this effect was enhanced when this cell line was cultured together with a tumor-associated macrophage line (>80% inhibition). This inhibition is due to cell cycle arrest in the G1 phase, and increased apoptosis mediated by caspase-3 activation [141].

Similar results were shown by Su et al. using crude garlic extract at 1 µg/mL in a human colon adenocarcinoma line (Colo205) [142]. However, in a similar study with HT-29 colon adenocarcinoma cells, no cytotoxic effect was found until 1 mg/mL of treatment concentration was used [143]. In this sense, the extraction method as well as the concentration of organosulfur compounds present in these extracts may play a fundamental role in obtaining successful results.

In turn, anticancerogenic effects against CRC have been found in other *Allium* vegetables as well. For example, *Allium subhirsutum* (popularly known as hairy garlic) is known to have concentrations above 100 mg/g of sulfur compounds. Extracts of this *Allium* species have been found to be highly effective in vitro against CRC with an IC_50_ of 71 µg/mL against the colon carcinoma line HCT116 [144]. It has also been shown that *Allium victorialis* var. *platyphyllum* (known as Myung-I in Korea) extract at a concentration of 0.2 mg/mL reduced cell viability by 68% in the HT-29 cell line. In addition, the antimetastatic activity of the extract has even been observed when it is administered intraperitoneally at 1 mg/kg of body weight in a mouse model of CRC metastasis in the lungs [145].

An interesting effect of garlic extracts is the adjuvant effect when administered together with classical chemotherapy treatments such as 5-Fluorouracil or oxaliplatin. In this regard, studies by Ortiz et al. showed a synergistic inhibitory effect over 60% in Caco-2 and HT-29 cell lines when these chemotherapies were used in combination with garlic extract concentrations of up to 200 µg/mL. The use of these combinatorial treatments can reduce the economic cost by 45.3% compared to the use of a single chemotherapy agent, so their implementation could be helpful to healthcare systems [146].

#### 3.2.1. Aged Garlic Extract

Aged garlic extract is a preparation made from fresh garlic that has been preserved for at least 10 months in an ethanol solution at room temperature. Through this process, the garlic components, such as flavonoids, S-allycysteine, pyruvate, benzyl cysteine compounds, lipid-soluble allyl sulfides, nutrient saponins, etc., are concentrated compared to fresh garlic [147]. In vitro studies by Dong et al. showed a cytotoxic effect of aged garlic extract (AGE) on the CRC cell line HT-29. Concentrations of 100 mg/mL of AGE were able to suppress cell growth by up to 64%. This effect is caused by an induction of G0/G1 cell cycle arrest, because of a downregulation of the PI3K/Akt pathway involved in proliferation, migration, and apoptosis [148]. These data were corroborated by Matsura et al. on different CRC lines such as HT-29, Sw480, and Sw620, where concentrations as low as 0.1 mg/mL significantly reduced proliferation. In addition, in their studies using AGE, they observed an anti-angiogenic effect. When AGE was added to endothelial cell lines (ECV304 and TRLECs cells) at a concentration of 10 g/L, an increase in cell adhesion and a reduction in mortality and invasion were observed, preventing the growth and development of cancer cells [149].

Similar results were obtained in studies using CRC rat models with dimethylhydrazine. Katsuki et al. fed the animals with AGE at 4% and observed a reduction in adenomas in the small intestine and a lower number of aberrant crypt foci. Jikihara et al. confirmed these studies with very similar effects when AGE at 3% was administered. In both studies, they found a reduced expression of cell proliferation markers (such as PCNA or MIB-5) and an attenuation of NF-κB activity, concluding that AGE may act as a chemopreventive agent with a suppressive effect on cell proliferation [150,151].

Finally, an interventional study by Tanaka et al. in patients with colorectal adenomas demonstrated promising results. In this study, each patient was administered 2.4 mL of AGE daily for 12 months. Only 37 patients completed this study, but it can be seen that in the group administered with AGE, the number and size of the adenomas was reduced, while in the control group, both values were increased [152]. In this study, the AGE preparation protocol and garlic concentration are not presented.

#### 3.2.2. *Allium roseum* L. var. Grandiflorum Briq. Essential Oil

Considering all the investigations into the anticancer properties of garlic and its extracts, it is surprising that so little has been researched involving garlic oils. Numerous lipophilic molecules identified from garlic have been demonstrated to have antitumoral properties (allicin, DAS, DADS, DATS, etc.) and these molecules individually have received far more attention than intact garlic oil. One study of *Allium roseum* bulb essential oil demonstrated its antiproliferative effects in two CRC cell lines. They found that culturing both HT-29 and Caco-2 cells with 4 and 8 µg/mL of the oil, respectively, was sufficient to reduce cell viability by 50%, while culturing with 20 µg/mL caused 100% cell death in both lines. They also found that the oil had potent antioxidant activities and significantly reduced ROS production in both lines as well [153]. This study should be repeated using healthy colonic cells to ensure that the antiproliferative effects are cancer-cell specific.

### 3.3. Bioactive Molecules

#### 3.3.1. Allicin

Allicin, as one of the primary metabolites of crushed garlic, has tremendous potential as a dietary supplement. Furthermore, its potential in CRC treatment and prevention has been extensively studied. One study of allicin’s effects on CRC cells in vitro found that it significantly reduced cell viability in a dose-dependent manner in an HT-29 cell line model, with an IC 50 of 37.5 µM (Table 1). They also found that it reduced cell proliferation and induced both of these effects through a decrease in intracellular glutathione levels, and an increase in the formation of reactive oxygen species (ROS) [98]. A second study confirmed the findings that allicin treatment reduces cell proliferation and viability, with an IC 50 of around 25 µM, but also found that allicin-treated cells had significantly reduced levels of phosphorylated STAT3 [99]. STAT3 is a transcription factor for genes involved in survival and proliferation that must be phosphorylated to initiate its activity. Thus, preventing STAT3 phosphorylation is one of the ways that allicin can reduce tumor cell viability. Incredibly, a third study found that allicin treatment was even more effective in reducing DLD-1 cell line viability than treatment with 5-Fluorouracil, a commonly used chemotherapeutic agent, with an IC 50 near 50 µM (Table 1). Furthermore, they found that allicin was less cytotoxic to healthy cells than 5-Fluorouracil, demonstrating both its effectiveness against CRC cells as well as the reduced likelihood of negative side effects [100]. On top of allicin’s antiproliferative activity and its selectivity for tumor tissues, Huang et al. also found that allicin treatment increased the radiosensitivity of HCT116 CRC cells and inhibited their migration ability as tested by a transwell chamber assay at 4 µg/mL [101]. Thus, allicin shows great potential as a pre-radiation therapy chemotherapeutic agent.

Beyond allicin’s promise in vitro, several studies have also confirmed its antitumoral effects in vivo. Huang et al. further confirmed its in vitro effects in a BALB/c mouse model with CT-26 cell-derived xenografts. They found that combination therapy of intraperitoneally injected allicin at 5 mg/kg of body weight and radiation significantly reduced the tumor weight compared to allicin treatment or radiation therapy alone [101]. With this said, it should be noted that the allicin and radiation dosage given in the combination treatment was equal to the doses used in the individual treatments. As such, rather than allicin improving the radiosensitivity of the CRC cells, it could be argued that allicin reduces cell viability in a complementary way to radiation therapy and so mixing two complementary treatments is always likely to achieve augmented results. It would be interesting to see if combining reduced doses of allicin and radiation therapy could achieve similar results, as this could reduce the negative side effects associated with chemo and radiation therapies.

Furthermore, allicin has also been demonstrated to function as a chemopreventive agent in an azoxymethane/dextran sodium sulfate (AOM/DSS)-induced CRC mouse model. It was found that dietary allicin supplementation at 48 mg/kg of feed significantly reduced tumor size and incidence compared to controls. The allicin-supplemented mice also recovered significantly more quickly from DSS-induced inflammation than did the un-supplemented controls [99]. This supports findings that associate high garlic intake with a reduced risk of CRC development and indicates that allicin is likely one of the bioactive compounds responsible for this effect. Furthermore, allicin’s anti-inflammatory properties could make it particularly useful in the chemoprevention of colitis-associated CRC.

#### 3.3.2. Alliin/S-Allyl-L-Cysteine Sulfoxide (SACS)

Alliin, also known by its chemical name S-Allyl-L-cysteine sulfoxide (SACS), is a major constituent of garlic that becomes rapidly metabolized into allicin when the garlic is crushed or minced. Because of this, it is not regularly consumed in the human diet and has received comparatively less attention than allicin and its derivatives as a potential treatment for CRC. Even so, one study conducted in silico analyses of the binding affinities of various organosulfur compounds toward epithelial growth factor receptor (EGFR) using the CDOCKER software version DS 4.0. They found that, of the compounds used in the investigation, only alliin was predicted to interact with EGFR. They next confirmed the findings in vitro by treating HCT-15 cells with alliin and found that the treatment significantly reduced EGFR gene expression as well as cell viability (IC 50 approximately 100 µg/mL) (Table 1). Furthermore, alliin was found to satisfy Lipinski’s rule of five and Veber’s protocol for drug-like properties and bioavailability [102].

Numerous in vivo studies have further confirmed alliin’s antitumorigenic properties. One study in F344 rats by Hatono et al. found that dietary alliin supplementation at 125 to 250 mg/kg of feed before and during cancer induction significantly reduced the formation of aberrant crypt foci. This effect was not repeated when alliin supplementation began after cancer induction [103], indicating alliin’s chemopreventive, rather than chemotherapeutic, potential. This effect was further confirmed in a second study with dietary supplementation at 125 mg/kg of feed (Table 1) [104]. Furthermore, a separate study found that oral administration of alliin by gavage in C57BI/6J mice at a dosage of 200 mg/kg of body weight significantly reduced nuclear damage in colonic mucosal cells. This result was followed up by confirmation that similar alliin administration significantly reduced the frequency of colonic tumors in CF-1 mice. Interestingly, although numerous organosulfur compounds were tested in this study (DAS, DADS, DPS, DPDS, ajoene, SAC, SPC, and SAMC), only alliin (SAC) was shown to be protective of nuclear damage [105].

The mixture of in silico, in vitro, and in vivo results indicates that alliin may have potential as a chemopreventive agent, but not likely as a chemotherapeutic agent. Unfortunately, the lack of in vitro studies limits our knowledge of its mechanism of action. Furthermore, all the in vivo studies that have been conducted on this topic were undertaken before the year 2000, and as such, these results would benefit from further confirmation using more modern techniques.

#### 3.3.3. Diallyl Sulfide (DAS)

Diallyl sulfide (DAS) is one of the most studied lipophilic, garlic-derived molecules. In vitro studies have demonstrated its ability to induce apoptosis and inhibit migration and metastasis in CRC cell lines. One study found that treating a CRC cell line (Colo-320 DM) with 50 µM DAS induced G2/M phase cell cycle arrest, increased ROS, and induced apoptosis via increased caspase-3 expression [106]. A separate study by Lai et al. found that DAS reduced Colo-205 CRC cell proliferation by inducing the downregulation of PI3K, Ras, MEKK3, MKK7, ERK1/2, JNK1/2, and p38. They also found that DAS treatment significantly reduced CRC cells’ migration and invasion capabilities via a reduction in matrix metalloproteinase-2 (MMP-2) expression. Although they found that DAS reduced proliferation and inhibited migration and invasion (IC 50 greater than 50 µM), they also found that both diallyl disulfide (DADS) and diallyl trisulfide (DATS) were even more effective in reducing proliferation and inhibiting MMPs (IC 50 25 µM for both) (Table 1) [107]. Interestingly, another study found that DAS treatment of Colo-205 CRC cells caused a decrease in multidrug resistance 1, 3, 4, and 6 proteins (MRP1, 3, 4, 6), indicating that DAS may be particularly useful in co-treatments [108].

In contrast to the positive in vitro results, in vivo studies have yielded more mixed results. The same study that found that DAS treatment decreased MRP expression in cell lines also investigated DAS’s effectivity against a Colo-205 CRC cell line-derived xenograft in BALB/C mice via intraperitoneal injections of DAS at 6 mg/kg body weight (Table 1). They found that tumor size trended downward in DAS-treated mice, but that the results were not significant. Furthermore, in the in vivo models, DAS yielded a significant increase in Mdr1 expression, but no significant change in MRP1, 3, 4, or 6 expressions [108]. All of this indicates that the effects of DAS are significantly reduced in vivo. In this study, DAS was injected intraperitoneally, so poor bioavailability or increased liver metabolism could be the cause of the more limited efficacy.

A separate study testing the effects of dietary DAS supplementation in APC^min^ mice found that supplementation with 100 to 300 ppm reduced the incidence of colonic polyps in a dose-dependent manner, although the inhibition was not statistically significant [109]. The authors claim that the lack of statistical significance is likely due to the relatively small number of mice used. DAS may be more useful as a dietary supplement since it readily encounters the colonic mucosa in this way and thus its poor bioavailability is less problematic than what was observed in the xenograft model. Thus, it may have better functionality as a chemopreventive agent than as a chemotherapeutic agent.

#### 3.3.4. Diallyl Disulfide (DADS)

Diallyl disulfide (DADS) is one of the most extensively studied garlic-derived organosulfur compounds in terms of cancer treatment. Its effects in CRC models have been investigated in many in vivo and in vitro studies. Numerous studies have demonstrated DADS’ ability to reduce proliferation and induce apoptosis in CRC cell lines (SW620, SW480, HCT116, HT-29, DLD-1), with IC 50s ranging from 5 to 50 µM depending on treatment time and cell line (Table 1). Multiple mechanisms for this effect have been observed including the downregulation of RAC1 and BCL-2, overexpression of BAX, BAD, and TRAIL, reduced NF-κB nuclear localization, and inhibition of the PI3K/Akt pathway [110,111,112]. A separate study found that DADS treatment induced a release of intracellular calcium stores in SW480 cells. While apoptosis was not measured in this study, the buildup of intracellular calcium would cause endoplasmic reticulum and mitochondrial stress, which could lead to apoptosis via the intrinsic pathway [113].

Beyond its cell viability-reducing effects, DADS has also been found to inhibit migration and invasion. One group found that DADS treatment at 30 to 45 mg/L downregulated Rac1 expression, which reduced PAK1-LIMK1-Cofilins signaling and inhibited the epithelial–mesenchymal transition in SW620 and HT-29 cells, as measured by transwell chamber and wound healing assays (Table 1) [110]. The same study further confirmed these results in vivo by measuring a reduced number of pulmonary metastatic nodules of nude mice that had HT-29 cells injected into their tail veins and received intraperitoneal injections of DADS at 100 mg/kg of body weight. A second study found that DADS treatment at 45 mg/L reduced LIMK1 expression, which reduced destrin and cofilin phosphorylation. This significantly reduced cell migration and invasion as measured by a scratch wound assay [114]. A third study found that DADS treatment at 25 µM induced the downregulation of MMP-2 and 7, as well as RAS, MEKK3, JNK1 and 2, and COX-1 and 2. It also reduced cell migration and invasion more effectively than treatment with diallyl sulfide (DAS) [107]. Also, much like diallyl sulfide (DAS), DADS treatment significantly reduced the expression of MRP1, 3, 4, and 6 in cell culture, indicating its potential in co-treatments [108].

In contrast to diallyl sulfide (DAS), DADS treatment has yielded numerous positive results in in vivo studies. DADS treatment via intraperitoneal injection at 6 mg/kg in BALB/c mice with Colo-205 cell-derived xenografts yielded a significant reduction in xenograft size (Table 1). This is in contrast with DAS, which yielded a downward trend in xenograft size without reaching statistical significance. Furthermore, in contrast to the in vitro studies, gene expression analysis demonstrated that DADS treatment in vivo caused an upregulation of Mdr1, MRP1, MRP4, and MRP6 drug resistance proteins [108]. Thus, while DADS may be more effective than DAS in reducing tumor size, it may not be useful in co-treatments. A second study found that regular DADS treatment, administered intraperitoneally at 100 mg/kg body weight, significantly reduced the size of cell-derived xenografts in BALB/c nude mice. Similarly, to what was found in the in vitro studies, LIMK1 overexpression reduced the effectiveness of the DADS treatment [114]. In contrast to these results, a separate study found that DADS treatment alone did not reduce the size of cell-derived xenografts in BALB/c nude mice. They reported that DADS was only effective against xenografts made of TRAIL overexpressing cells [111]. This study did not specify how the DADS was administered nor its dosage, so it is impossible to compare it with the other studies. Finally, the chemopreventive properties of DADS were investigated in a study where FVB/N mice were fed diets supplemented with 42 ppm DADS and had CRC chemically induced by azoxymethane (AOM) and dextran sodium sulfate (DSS). They found that DADS supplementation significantly reduced tumor incidence, number, and burden when compared with mice fed standard diets. Furthermore, they found that the mice eating the supplemented diet recovered from DSS-induced inflammation much more rapidly than those eating the standard diet [112].

DADS shows tremendous promise as a potential chemotherapeutic agent due to its antiproliferative and antimetastatic properties, as demonstrated both in vitro and in vivo. It also shows great potential as a chemopreventive agent that could be used as a dietary supplement. For these reasons, DADS should be further studied, both in vivo and potentially in clinical human trials. Interestingly, as of writing, no clinical trials of any sort have involved DADS, as per ClinicalTrials.gov; thus, it is likely that further pre-clinical studies are necessary.

A major potential downside to DADS is its hydrophobicity. This gives it low bioavailability and severely limits its use in clinical settings. Several studies have attempted to circumvent this problem and increase DADS’ aqueous solubility by encasing it in liposomes [115] or nanoparticles [116]. These techniques have proven fruitful in in vitro studies, with both modifications achieving greater reductions in CRC cell viability and increased intracellular DADS concentrations. As of yet, these studies have not been repeated in vivo.

#### 3.3.5. Diallyl Trisulfide (DATS)

Diallyl trisulfide (DATS) has received comparatively less attention as a potential CRC chemotherapeutic than DADS has, yet it has been proven effective against CRC models both in vitro and in vivo. In vitro, two recent studies have demonstrated that treating SW480 and DLD-1 cell lines, as well as patient-derived primary CRC cells, with DATS induced apoptosis as observed by the increased expression of proapoptotic proteins, DNA condensation, and increased reactive oxygen species with IC 50s ranging from 30 to 40 µM depending on the cell type (Table 1) [117,118]. Furthermore, it has been shown to be effective against CRC stem cells at 40 µM, which are normally particularly difficult to treat. One study found that treating CRC stem cells derived from SW480 and DLD-1 cell lines with DATS inhibited the Wnt/β-catenin pathway and reduced colonosphere formation [117]. This effectiveness against CRC stem cells may be partially because DATS, much like DADS and DAS, significantly reduced the expression of multidrug resistance-associated proteins (MRP 1, 3, 4, 6) in CRC cell culture [108]. Cancer stem cells are known to exhibit increased levels of multidrug resistance-associated proteins [119], and so a decrease in their expression induced by DATS could reduce their chemoresistance. Furthermore, DATS, much like DADS and DAS, has been demonstrated to reduce migration and invasion capabilities at concentrations as low as 10 µM, as measured by transwell chamber and wound healing assays. In contrast to DAS and DADS, DATS was observed to induce the greatest antimetastatic effects [107].

DATS has also shown positive results in numerous rodent CRC models. Two studies of cell-derived xenografts (Colo-205, CT-29) in BALB/c mice have shown that regular treatment with DATS, either by gavage at 50 mg/kg body weight or by intraperitoneal injection at 6 mg/kg body weight, significantly reduced the size and weight of the xenografted tumors when compared to saline-treated controls (Table 1) [108,120]. Similarly, to what was observed with DADS treatment, DATS-treated xenografts showed significantly increased expression of MRP 1, 4, and 6. Once again, this contrasts directly with the results observed in cell lines [108]. Furthermore, these results call into question the validity of the anti-stem cell effects that were observed in cell lines.

DATS has also been demonstrated to be effective as a chemopreventive agent against CRC. One study found that DATS treatment at a concentration of 25 mg/kg body weight, three times per week via an unspecified route, significantly reduced tumor number and incidence in an AOM-induced CRC mouse model (Table 1) [23]. Much like DADS, DATS is also hydrophobic and exhibits low bioavailability, limiting its potential for clinical use. This same study formulated DATS in lipid nanoparticles to increase its bioavailability and found not only its effectiveness in the AOM model but also increased kinetics and greater efficacy at lower doses against RKO and HT-29 cell lines.

Due to its demonstrated efficacy in vitro and in vivo, DATS should be considered a strong candidate for further study and development as a chemotherapeutic agent against CRC. Its main limitations come from its limited bioavailability, but novel techniques such as encapsulation could be effective in circumventing these problems.

#### 3.3.6. Diallyl Tetrasulfide

In contrast to the other diallyl compounds discussed thus far in this review, diallyl tetrasulfide as a CRC chemotherapeutic has only been investigated in one single study. In SW480/620 and HT-29 cell lines, Yagdi et al. discovered that diallyl tetrasulfide is a reversible tubulin binder that induces mitotic arrest and apoptosis via the inhibition of autophagy at 10 to 50 µM (Table 1). p62 protein expression seems to protect the CRC cell lines against diallyl tetrasulfide-induced apoptosis since HT-29 cells contained greater amounts of p62 and were much more resistant than the other cell lines. They further confirmed that the diallyl tetrasulfide treatment of CRC cell lines significantly reduced spheroid formation. This effect was also cell-line-dependent (HT-29 cells were protected) and seemed to be mediated by levels of p62 expression since p62 knockdown sensitized the resistant cells. Furthermore, zebrafish implanted with HT-29 cell-derived xenografts from CRC cell lines experienced significantly less tumor growth when treated with 50 µM dibenzyl tetrasulfide (a synthetic derivative of diallyl tetrasulfide that shows similar effects in vitro) [121].

#### 3.3.7. Methylsulfonylmethane (MSM) ((Dimethyl Sulfone (DMSO2))

Methylsulfonylmethane (MSM) is synthesized by the oxidation of dimethyl sulfoxide (DMSO) and is found in a variety of vegetables [154]. Its activity against a variety of cancers has been investigated, but so far only one study has investigated its function as a chemotherapeutic agent against CRC. Kim et al. found that treating HT-29 cells with MSM reduced viability, induced cell cycle arrest at the G_0_/G_1_ checkpoint, and induced apoptosis with IC 50s of 250 mM at 24 h and 100 mM at 48 h (Table 1). The cell cycle arrest coincided with the decreased expression of cyclin D and E, CDK4, and Rb proteins, and apoptosis was induced via mitochondrial membrane disruption. Furthermore, MSM reduced the stemness of the CRC cell lines as measured by reduced sphere formation and downregulation of the typical stem cell markers SOX2, NANOG, and OCT4 [24].

Thus, MSM shows some potential as a chemotherapeutic agent against CRC, but the current results are very preliminary. The study discussed above was only conducted in one cell line, and as has been observed with many of the other molecules already discussed in this review, the results are often cell-line-dependent. As such, further experiments in other cell lines are necessary to confirm its usefulness. Furthermore, no in vivo studies have been conducted. Interestingly, MSM supplementation is already somewhat common amongst patients with various ailments, so this molecule may be particularly amenable to investigation in the clinic [154].

#### 3.3.8. (*Z*)-Ajoene

(*Z*)-ajoene is an organosulfur compound found in crushed garlic that has only recently begun to be investigated as a potential chemotherapeutic agent against CRC. In the only study to date investigating its impact, Li et al. found that (*Z*)-ajoene reduced the viability of SW480 CRC cells in a dose-dependent model, with an IC 50 of 30 µM at 72 h (Table 1). Furthermore, they found that (*Z*)-ajoene significantly reduced nuclear β-catenin protein levels, as well as the levels of proteins whose transcription is activated by the Wnt/β-catenin pathway (c-Myc and cyclin D1). It was further confirmed that (*Z*)-ajoene increases the phosphorylation of cytosolic β-catenin, which induces its degradation, and thus inhibits the Wnt/β-catenin pathway [122].

#### 3.3.9. S-Allylmercaptocysteine (SAMC)

S-allylmercaptocysteine (SAMC) is a non-volatile and soluble compound present in aged garlic. This compound can be extracted from garlic bulbs or produced by chemical synthesis from L-cysteine and allicin in an aqueous solution at pH 6 [123].

At different SAMC concentrations between 200 and 450 µM, numerous anticarcinogenic properties have been observed. These include the inhibition of cell proliferation via G2-M phase cell cycle arrest, the induction of apoptosis, and the reduction in invasion in different CRC lines such as HT-29, Sw-480, Sw-620, Caco-2, or HT116 [124,125,126,127]. The molecular mechanism that explains the induction of apoptosis is via the JNK and p38 pathways that activate a signaling cascade that ultimately leads to the activation of the tumor suppressor genes p53 and Bax [125].

In addition, a very promising effect of SAMC in combinatorial therapy has been observed. Tong et al. observed a synergistic effect on the induction of apoptosis when SAMC at 800 µM was used in combination with MAPK inhibitors. This effect is produced via the activation of the TGF-β pathway, which leads to the expression of apoptotic proteins such as ERK and JNK (Table 1) [127].

A similar effect has been seen by Li et al. when combining SAMC with a chemopreventive agent such as rapamycin, highlighting the potential adjuvant functions of this compound. In an HCT116 xenograft mouse model, they observed an 80% reduction in tumor growth with this combinatory therapy of oral SAMC administration at 300 mg/kg body weight/day with intraperitoneal injections of rapamycin at 5 mg/kg body weight/day (Table 1). This effect has been shown to be due not only to the induction of apoptosis but also to the activation of autophagy and antioxidant responses via Nrf2 [124].

Finally, it has been shown that SAMC may function as a good agent against CRC metastasis, probably by restoring the expression of E-cadherin levels in Caco-2, SW480, and SW620 cells at a 400 µM concentration [126].

## 4. Conclusions

Organosulfur compounds show great promise in colorectal cancer prevention, but many questions remain. *Allium* and cruciferous vegetable extracts have shown interesting results in human studies, but complete clinical trials remain to be conducted. For individual molecules, sulforaphane, phenethyl isothiocyanate, 3,3′-diindolylmethane, allicin, and diallyl disulfide have been extensively studied in multiple cell lines and animal models but lack studies in humans. These molecules are effective at physiological doses but require optimized administration. In the case of sulforaphane, intraperitoneal administration to treat xenografts in mice is about 100 times more potent than the tested oral administration (14.1 μg versus 1600 μg per day, respectively). In the case of phenethyl isothiocyanate, 0.8 mg per day per mouse orally reduced the tumor size in a xenograft model, in comparison with 2.4 mg per mouse intraperitoneally as chemoprevention. In the case of 3,3′-diindolylmethane, 1.6 mg per mouse intraperitoneally enhances the xenograft’s sensitivity to 5-fluorouracil (but not in an *APC* mouse model), whereas orally, this same dosage caused positive effects in the absence of the drug. In the case of allicin, 0.2 mg per day in mice has chemopreventative properties, in comparison to a mixture of a radiation treatment plus 0.2 mg per day of allicin (intraperitoneal administration) to reduce tumor size. In the case of diallyl disulfide, 0.24 mg per mouse intraperitoneally reduced xenograft size, whereas 0.16 mg per day orally in mice was effective in chemoprevention.

Major limitations for the clinical use of these organosulfur compounds include the difficulty in comparing the achieved bioactivity among the different possible administration routes (gavage, diet, encapsulated, intraperitoneal, in co-therapy, etc.), as well as current data from diverse animal species and lineages. Pharmacokinetic data in humans are unknown for some of these compounds under the desired administration routes, a key limitation for their clinical development as antitumors. These data are needed for selecting the most useful dosage, as well as data from studies including side effects monitoring. Furthermore, the industrial supply for some of these compounds must be secured at a large scale. Finally, clinical trials will be needed to confirm whether any of these molecules are more effective than the current standard of care in humans.

The remaining molecules are not yet candidates for clinical studies due to their lack of investigation in pre-clinical models. Benzyl isothiocyanate has been extensively investigated in cell lines and animal models but requires follow-up studies to address unanswered questions. Other molecules, such as sulforaphene, indole-3-carbinol, diallyl sulfide, and S-allylmercaptocysteine, have been proven effective in cellular models but require confirmation from studies in animal models. The remaining molecules, including alliin, iberin, allyl isothiocyanate, diallyl tetrasulfide, (*Z*)-ajoene, *Allium roseum* oil, and methylsulfonylmethane, are still in their infancies in terms of CRC investigation. Many of these would benefit from confirmation in a wider variety of cell lines and more in-depth mechanistic studies.

As a final conclusion, in vitro and animal in vivo tests show that the organosulfur compounds with the highest future potential for use at the clinical level are sulforaphane, phenethyl isothiocyanate, 3,3′-diindolylmethane, allicin, and diallyl disulfide. These have been most extensively studied and demonstrated in animal models for colon cancer and show efficacy at reasonable doses.

## Figures and Tables

**Table 1:** Summary of bioactive-tested concentrations for the different organosulfur compounds in in vitro and in vivo assays.

Compound	Summary	Positive Dosing	Negative Dosing	Citations
Sulforaphane	Induced cell cycle arrest and apoptosis, and reduced angiogenesis and migration in vitro. Reduced xenograft size and tumor initiation in vivo.	7–60 µM in cell culture, 0.08 µmoles via daily intraperitoneal injection, 300 ppm in diet (1.6 mg/day).		[47,48,49,55,56,57,58,59,60,61,62,63,64,65,66,67,68,69,70,71,72,73]
Sulforaphene	Induced cell cycle arrest at the G2/M phase, upregulation of the JNK pathway, inhibition of microtubule polymerization, and increase in intracellular reactive oxygen species in vitro. Reduced xenograft size in vivo.	5 µM in cell culture, 5 mg/kg of body weight daily intraperitoneal injection.		[74,75]
Allyl isothiocyanate (AITC)	Induced cell cycle arrest and apoptosis, and reduced migration in vitro.	10–20 µM in cell culture.		[76,77]
Phenethyl isothiocyanate (PEITC)	Reduced viability, stemness, and spheroid formation in vitro. Reduced xenograft size, and tumor initiation in vivo.	12–88 µM in vitro, 60 mg/kg of body weight daily intraperitoneal injection, and 20 mg/kg of body weight daily oral administration.		[78,79,80]
Benzyl isothiocyanate (BITC)	Induced cell cycle arrest and apoptosis in vitro. Increased tumor initiation in vivo.	10–20 µM in cell culture.	0.5 g/kg of diet	[81,82,83]
Iberin	Reduced proliferation, methyl guanine methyl transferase methylation, and increased miRNA expression in vitro.	8–10 µM in cell culture.		[84,85]
Indole-3-carbinol	Reduced viability and proliferation, plus p53 upregulation in vitro. Both reduced and increased tumor initiation in vivo.	500 µM to 1 mM in cell culture, 1 g/kg of diet.	1 g/kg of diet.	[65,86,87,88,89,90,91]
3,3′-Diindolylmethane	Reduced viability, cell cycle arrest, and COX1/2 and ERK1/2 protein inhibition in vitro. Reduced xenograft size in vivo.	25–56 µM in cell culture, 40 mg/kg of body weight via intraperitoneal injection.	40 mg/kg of body weight via oral administration.	[92,93,94,95,96,97]
Allicin	Reduced viability, proliferation, and migration in vitro. Reduced xenograft size and tumor initiation in vivo.	25–50 µM for viability and 4 µg/mL for migration in cell culture. Intraperitoneal injection of 5 mg/kg of body weight and 48 mg/kg of diet.		[98,99,100,101]
Alliin/S-Allyl-L-cysteine sulfoxide (SACS)	Reduced viability and EGFR (epithelial growth factor receptor) expression in vitro. Reduced tumor initiation in vivo.	100 µg/mL in cell culture, 125 mg/kg of diet, 200 mg/kg of body weight administered orally.		[102,103,104,105]
Diallyl sulfide (DAS)	Induced apoptosis and inhibited migration/metastasis in vitro. Non-significant reductions in xenograft size and tumor initiation in vivo.	50 µM in cell culture.	Intraperitoneal injection of 6 mg/kg of body weight and 300 ppm in diet.	[106,107,108,109]
Diallyl disulfide (DADS)	Reduced viability, migration, and invasion, and increased apoptosis in vitro. Reduced metastasis, xenograft size (best in TRAIL overexpressing tumors), and tumor initiation in vivo.	5–50 µM for viability and 25 µM for migration in cell culture. Intraperitoneal injection of 100 mg/kg of body weight and 42 ppm in diet.		[107,108,109,110,111,112,113,114,115,116]
Diallyl trisulfide (DATS)	Induced apoptosis, reduced stem cell viability, and reduced migration/invasion in vitro. Reduced xenograft size and tumor initiation in vivo.	30–40 µM for apoptosis, 40 µM for stem cells, 10 µM for migration. Intraperitoneal injection of 6 mg/kg of body weight and oral administration of 50 mg/kg of body weight.		[23,107,108,117,118,119,120]
Diallyl tetrasulfide	Induced cell cycle arrest, apoptosis, and reduced spheroid formation in vitro. Modified molecule reduced xenograft size in vivo.	10–50 µM in cell culture. An amount of 50 µM dibenzyl tetrasulfide in zebrafish.		[121]
Methylsulfonylmethane (MSM) ((dimethyl sulfone (DMSO2))	Reduced viability, stemness, and spheroid formation in vitro.	100–250 mM in cell culture.		[24]
(Z)-ajoene	Reduced viability and Wnt/β-catenin pathway inhibition in vitro.	30 µM for 72 h of treatment.		[122]
S-allylmercaptocysteine (SAMC)	Reduced proliferation, induced apoptosis, and reduced migration in vitro. Reduced xenograft size in combination treatment in vivo.	200–450 µM for apoptosis, 400 µM for migration, and 300 mg/kg body weight/day administered orally in combination with rapamycin treatment.		[123,124,125,126,127]
